# Individualized VMAT lattice radiotherapy on a standard flattening-filter linear accelerator for advanced bulky tumors: a retrospective feasibility and early clinical experience

**DOI:** 10.3389/fonc.2026.1926882

**Published:** 2026-08-11

**Authors:** Jingna Ji, Min Xiao, Yan Ding, Gangyang Yu, Qian Wang, Jing Xie, Lu Zhang

**Affiliations:** Department of Radiation Oncology, Hankou Hospital Affiliated with Wuhan University of Science and Technology (Wuhan Hankou Hospital), Wuhan, Hubei, China

**Keywords:** advanced bulky tumors, flattening filter mode, lattice radiotherapy, spatially fractionated radiotherapy, symptom palliation, technical feasibility

## Abstract

**Background:**

Advanced bulky tumors cause pain, hemorrhage, and functional impairment, but their volume, hypoxic core, and proximity to organs at risk limit dose escalation with conventional radiotherapy. Lattice radiotherapy (LRT) delivers ablative doses to intratumoral vertices while sparing peripheral tissue. We evaluated personalized LRT on standard flattening−filter (FF) linacs, alone or with radiotherapy.

**Methods:**

Seven patients (8 sites) with advanced bulky tumors treated February–November 2025 using 6 MV VMAT in FF mode were retrospectively reviewed. LRT (1–5 fractions of 6–15 Gy) was individualized by performance status, prior therapy, and tumor geometry; additional radiotherapy was at physician discretion. Co-primary endpoints were symptom relief (NRS, analgesic use, tumor-related symptoms) and local disease control; acute toxicity was secondary.

**Results:**

Median age was 69 years, median ECOG 2 (1–3), and mean tumor volume 370.7 cc (101.8–792.4 cc). All patients completed planned LRT. Symptom improvement occurred in 6 of 8 sites (75%), usually within two weeks. At median follow-up of 8 months (4–12), partial response (PR) was achieved in 5 of 8 sites (62.5%), stable disease (SD) in 2 (25.0%), and progressive disease (PD) in 1 (12.5%); the disease control rate (DCR = CR + PR + SD) was 7/8 (87.5%), with no complete response (CR) observed. Acute toxicity occurred in 3 patients (all grade 1–2, mainly fatigue and mild dermatitis); no grade ≥3 events occurred.

**Conclusion:**

Personalized LRT on FF linacs was feasible and well tolerated, with encouraging early palliative benefit in this small, heterogeneous cohort, supporting prospective evaluation with standardized symptom-assessment tools.

## 1 Introduction

Advanced bulky solid tumors frequently cause pain, functional impairment, and hemorrhage through local compression and invasion of adjacent structures. Because these patients typically present with advanced disease stage and poor performance status, surgical resection is often not feasible. Hypofractionated palliative radiotherapy (e.g., 20 Gy in 4–5 fractions or 30 Gy in 10 fractions) can provide a degree of symptom relief (1). However, for tumors with a diameter >=5 cm, disorganized intratumoral vasculature and insufficient perfusion generate hypoxic cores that substantially reduce radiosensitivity (2–4). In addition, the radiation tolerance of normal tissues declines as the irradiated volume increases, which constrains dose escalation in conventional external beam radiotherapy (EBRT) (5). Proximity of large tumors to surrounding organs at risk (OARs) further complicates treatment planning and limits the safe application of stereotactic body radiation therapy (SBRT) (6, 7).

Spatially fractionated radiation therapy (SFRT) addresses these challenges by partitioning the tumor volume into multiple small fields with steep dose gradients, delivering ablative peak doses while maintaining low valley doses in between (8–10). Over the past decade, the two-dimensional GRID technique has evolved into a three-dimensional configuration known as lattice radiation therapy (LRT), in which high doses are concentrated within intratumoral vertex regions while the periphery of the gross tumor volume (GTV) receives a lower dose. Several clinical series - predominantly single-arm and small-sample reports - have suggested that LRT is feasible across a range of bulky tumor types, with promising local control and an acceptable acute toxicity profile (11–13). Most of these reports, however, were based on flattening-filter-free (FFF) beams (14, 15), and several practical questions remain unresolved, including the optimal dose-fractionation scheme, the sequencing of LRT with conventional EBRT, and whether the technique can be reliably implemented on widely available standard flattening-filter (FF) linear accelerators.

To explore these questions, we retrospectively reviewed 7 patients (8 treatment sites) with advanced bulky tumors who received individualized LRT delivered on standard FF-mode linacs, either alone or combined with conventional radiotherapy. The present analysis focuses on technical feasibility, symptom palliation, treatment tolerability, and early disease control in this vulnerable patient population.

## 2 Materials and methods

### 2.1 Study design and patients

We retrospectively reviewed 7 patients with 8 distinct treatment sites who received lattice radiotherapy (LRT) at our center between February 2025 and November 2025. Eligibility criteria were: (1) histologically confirmed locally advanced or metastatic solid tumor with a maximal tumor diameter >=5 cm; (2) age >18 years; (3) ECOG performance status 0-3 - recognizing that patients with ECOG 3 were included when, in the treating physician’s judgment, the tumor-related symptom burden warranted a short, low-toxicity palliative regimen and the patient could tolerate lying still for treatment; (4) estimated life expectancy >3 months; and (5) unsuitability for, or refusal of, surgical resection. The study was approved by the Institutional Ethics Committee. Written informed consent for treatment was obtained from all patients prior to LRT; the requirement for written informed consent for publication was waived owing to the retrospective design. Baseline characteristics are summarized in **Table 1**.

**Table 1:** Baseline characteristics, treatment, and outcomes of the 7 patients (8 treatment sites).

No.	1	2	3	4-1	4-2	5	6	7
Sex	M	M	M	M	–	M	M	F
Age	75	77	62	68	–	75	69	57
ECOG	1	2	1	2	–	2	3	3
Stage	IV	IV	IV	IV	–	IV	IV	IV
Pathological Type	Moderately differentiated HCC	Tongue SCC, Grade II	Hypopharyngeal SCC	Renal extraskeletal osteosarcoma	–	Ureteral carcinosarcoma	Gallbladder mucinous adenocarcinoma	Lung adenocarcinoma
Prior RT	No	No	No	No	–	Yes	No	No
RT Modality	Sequential LRT+EBRT	Sequential LRT+EBRT	LRT boost+EBRT	LRT alone	LRT alone	LRT alone	LRT boost+EBRT	Sequential LRT+EBRT
Systemic Therapy	Targeted + Immunotherapy	None	Chemotherapy (TP)	ADC (sac-TMT)	–	Immunotherapy	None	Targeted therapy
Symptom Relief	Yes	Yes	Yes	Yes	No	No	Yes	Yes
Follow-up (mo)	12	11	9	5	5	4	7	8
Response	PR	PR	PR	SD	PD	SD	PR	PR
Status	NED	NED	NED	DOD	–	DOID	DOID	DOID
Acute Toxicity	None	G2 dermatitis	G2 dermatitis; G1 mucositis	None	None	G1 gastroenteritis	G2 gastroenteritis	None

Patient 4 had two treatment sites (4–1 and 4-2); site 4–2 inherits the patient’s baseline characteristics (shown as dashes). ECOG, Eastern Cooperative Oncology Group performance status; HCC, hepatocellular carcinoma; SCC, squamous cell carcinoma; RT, radiotherapy; LRT, lattice radiotherapy; EBRT, external beam radiotherapy; Prior RT, prior radiotherapy at the treated site; ADC, antibody-drug conjugate; Systemic Therapy, concurrent systemic treatment during radiotherapy. Response assessed per RECIST v1.1: PR, partial response; SD, stable disease; PD, progressive disease. Status: NED, no evidence of disease; DOD, died of disease; DOID, died of other/intercurrent disease. Symptom relief: sustained >=2-point reduction in NRS pain score and/or resolution of tumor-related symptoms for >=1 month. Acute toxicity graded per CTCAE v5.0: G1, grade 1 (mild); G2, grade 2 (moderate); no grade >=3 events occurred.

### 2.2 Lattice target delineation

**Figure 1:**
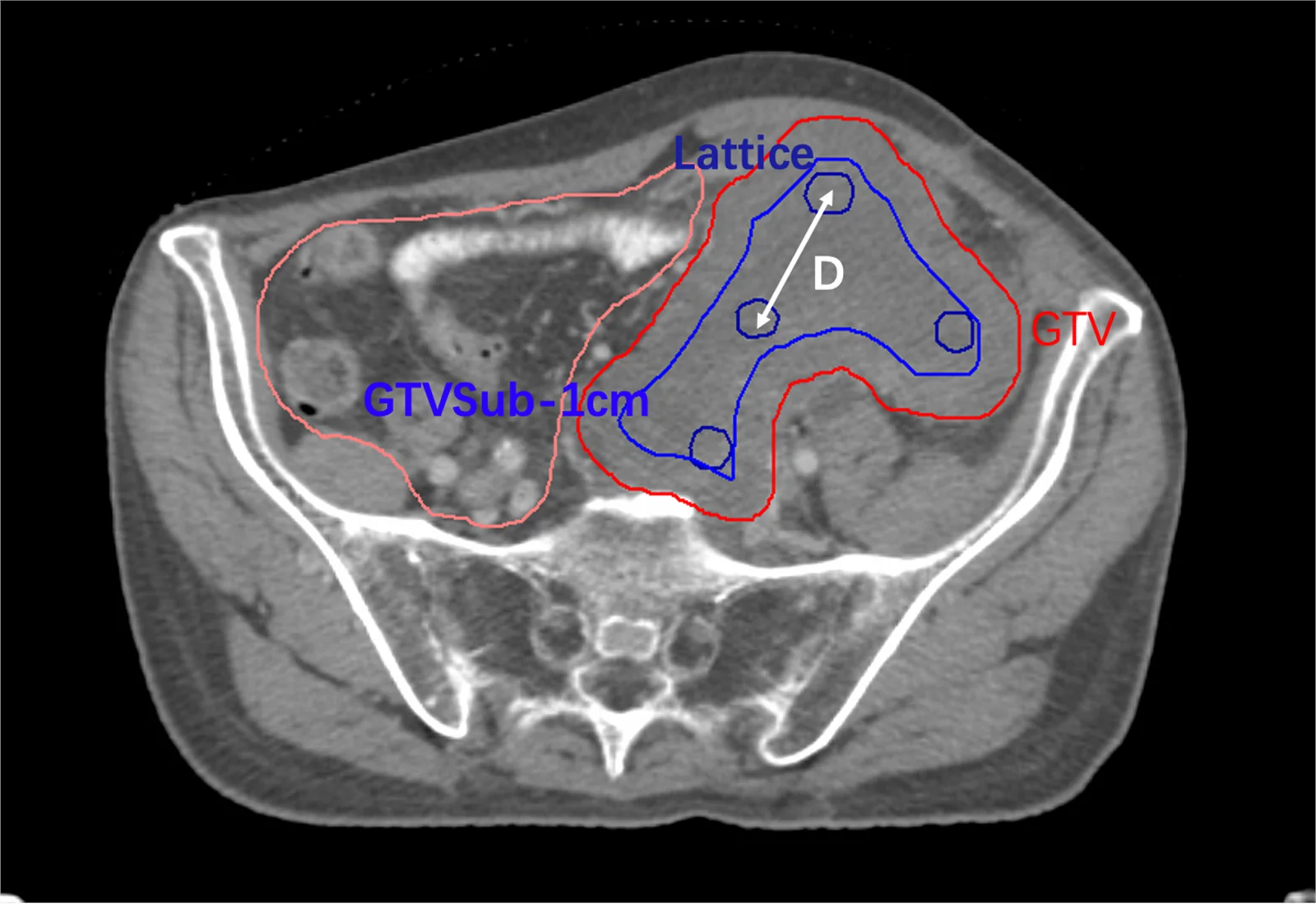
Schematic representation of the target volume and lattice configuration for a patient with ureteral sarcoma treated with Lattice Radiotherapy. D: The distance between the centers of two adjacent lattice spheres is 3–4 cm.

All patients underwent contrast-enhanced CT simulation according to standard radiotherapy protocols. The gross tumor volume (GTV) was delineated on simulation CT, fused with diagnostic MRI or CT where available. Lattice vertices (spherical high-dose regions) were placed according to the following institutional rules: (1) a GTVsub_1cm structure was generated by uniformly contracting the GTV by 1 cm, and all vertex spheres were confined within this structure and placed away from major vessels and nerves; (2) the Ablation Dose Ratio (ADR) - defined as the percentage of the GTV receiving ablative-dose hotspots - was maintained at 1%-10%; (3) the Lattice Density Index (LDI), defined as (number of vertex spheres/GTV volume) x 100, was targeted at 1%-8%; (4) the center-to-center distance (D) between adjacent vertices was 3–4 cm; and (5) each vertex sphere was 1.0-1.5 cm in diameter and positioned >=1.5 cm from any critical organ at risk (OAR). Target delineation and lattice configuration are illustrated in **Figure 1**.

### 2.3 Treatment planning, delivery, and treatment groups

All plans were generated on the Monaco treatment planning system (version 5.11) for an Elekta Synergy linear accelerator equipped with the Agility multileaf collimator (5 mm leaf width at isocenter). A 6 MV photon beam in flattening-filter (FF) mode was used at a dose rate of 600 MU/min. VMAT plans typically employed 3–4 arcs with non-coplanar collimator and couch angles tailored to target geometry and vertex distribution, in order to achieve steep dose falloff between the vertex peaks and the surrounding valley regions. Prior to delivery, every plan underwent 3D dosimetric verification using the OCTAVIUS 4D 1500 system (PTW), with a Gamma pass rate >=95% (3%/2 mm) as the acceptance criterion. OAR dose constraints followed AAPM Task Group 101 (16)and the 2018 UK consensus on normal-tissue dose constraints for SBRT (17), with the cumulative dose from both LRT and any combined EBRT taken into account.

The total beam-on time per fraction ranged from 4 minutes 15 seconds (P2) to 23 minutes 44 seconds (P4-1), with a mean of 11 minutes 42 seconds and a median of 9 minutes 31 seconds. Cone-beam computed tomography (CBCT) image guidance with soft-tissue matching was used for all treatment sites. For patients with abdominal tumors (P1 and P6), an abdominal compression plate was applied for respiratory motion management based on individual patient assessment. For P2, serial CBCT imaging revealed marked tumor regression with vertex spheres approaching the skin surface, prompting replanning between fractions to maintain target coverage while respecting skin dose constraints. All other patients completed treatment according to their original plans, as serial CBCT confirmed stable target position.

LRT was prescribed in 1–5 fractions of 6–15 Gy per fraction, individualized according to pathological type, anatomic site, ECOG status, prior radiotherapy history, and intended combination therapy. Because the cohort included patients with ECOG 0–3 and heterogeneous tumor types, dose prescriptions were individualized: patients with poorer performance status (ECOG 3) or previously irradiated sites were prioritized for shorter LRT-only or single-fraction regimens, whereas those with better performance status received integrated alternating LRT-EBRT. Based on the sequencing with EBRT, the 8 treatment sites were classified into three groups: (a) LRT alone (3 sites in 2 patients: sites 4-1, 4-2, and 5) — recurrent, previously irradiated cases in whom the cumulative dose to adjacent OARs (small bowel, stomach) precluded further EBRT. Single-fraction vertex doses of 10–20 Gy were delivered, consistent with the dose range reported in recent LRT systematic reviews (18, 19). (b) Single LRT boost followed by EBRT (2 sites: sites 3 and 6) — one LRT fraction (15 Gy) was delivered as a focal boost, followed by a conventionally fractionated or moderately hypofractionated EBRT course (54 Gy in 18 fractions and 39 Gy in 13 fractions, respectively). A vertex dose of 15–18 Gy × 1 fraction was delivered, followed by conventionally fractionated or moderately hypofractionated EBRT (20–66 Gy), following the boost paradigm reported by Ferini et al. (20) and Ahmed et al. (21). (c) Integrated alternating LRT-EBRT (3 sites: sites 1, 2, and 7) — an individualized multi-fraction vertex delivery scheme developed at our center, in which LRT fractions and EBRT fractions were delivered in alternating blocks: each LRT fraction was followed by a 2-day rest interval and then a block of approximately 5 consecutive daily EBRT fractions, with this cycle repeated 2–5 times according to individual tumor characteristics and overall treatment intent. This alternating scheme was informed by the multi-fraction LRT platforms reported by Xu et al. (22), and was adapted to our institutional workflow.

Five of the seven patients received concurrent systemic therapy, with all doses timed within the LRT treatment window to maximize spatiotemporal overlap: docetaxel plus cisplatin (TP) for P3 (hypopharyngeal SCC), sintilimab plus bevacizumab for P1 (HCC), sacituzumab tirumotecan for P4 (renal extraskeletal osteosarcoma), tislelizumab for P5 (ureteral carcinosarcoma), and dabrafenib plus trametinib for P7 (BRAF V600E-mutant lung adenocarcinoma); regimens were selected per current CSCO and NCCN guidelines for each histology, and full doses were maintained during LRT without modification (detailed in **Table 2**). Patients P2 (Recurrent tongue cancer after postoperative chemoimmunotherapy, followed by sequential radiotherapy) and P6 (gallbladder carcinoma with gastrointestinal obstruction precluding chemotherapy) did not receive concurrent systemic therapy.

**Table 2:** Concurrent systemic therapy regimens and best response in patients treated with VMAT lattice radiotherapy.

Patient	Primary diagnosis	Systemic therapy dose & schedule	Timing vs. LRT	Best response
1	Moderately differentiated HCC	Sintilimab 200 mgd1+ Bevacizumab 600 mgd1 q3w	Concurrent	PR
2	Tongue SCC, Grade II	None (Recurrent tongue cancer after postoperative chemoimmunotherapy, followed by sequential radiotherapy)	Sequential	PR
3	Hypopharyngeal SCC	Docetaxel 120 mg d1+ Cisplatin40 mg d1–3 q3w	Concurrent	PR
4-1	Renal extraskeletal osteosarcoma	Sacituzumab tirumotecan 200 mg d1 q3w	Concurrent	SD
4-2	Renal extraskeletal osteosarcoma	Sacituzumab tirumotecan 200 mg d1 q3w	Concurrent	PD
5	Ureteral carcinosarcoma	Tislelizumab 200 mg q3w	Concurrent	SD
6	Gallbladder mucinous adenocarcinoma	None (Gastrointestinal obstruction; chemotherapy was not administered.)	–	PR
7	Lung adenocarcinoma (BRAF V600E)	Dabrafenib 150 mg bid+ Trametinib 2 mg qd	Concurrent	PR

LRT, lattice radiotherapy; HCC, hepatocellular carcinoma; SCC, squamous cell carcinoma; q3w, every 3 weeks; bid, twice daily; qd, once daily; PR, partial response; SD, stable disease; PD, progressive disease (RECIST v1.1).

### 2.4 Follow-up and outcome assessment

Patients were evaluated at baseline, weekly during treatment, and at scheduled follow-up visits at 1 month, 3 months, and every 2–3 months thereafter until last follow-up or death, through outpatient review and telephone contact. The median follow-up was 8 months (range 4–12 months).

Symptom relief (co-primary endpoint) was assessed by the treating physician using the Numerical Rating Scale (NRS, 0-10) for pain intensity, supplemented by review of analgesic consumption and changes in tumor-related symptoms (bleeding, compression-related functional impairment) documented in the medical record. Symptom relief was defined as a sustained >=2-point reduction in NRS pain score, and/or a reduced analgesic requirement, and/or resolution of bleeding or pressure symptoms, maintained for at least one month without concomitant analgesic escalation.

Local disease control (co-primary endpoint) was defined as the absence of progressive disease per the Response Evaluation Criteria in Solid Tumors (RECIST) version 1.1 at last follow-up. Acute toxicity (secondary endpoint) was graded according to the Common Terminology Criteria for Adverse Events (CTCAE) version 5.0. The Tumor Reduction Rate (TRR), defined as TRR = (GTV_baseline - GTV_follow-up)/GTV_baseline, where GTV_follow-up was re-contoured by the treating physician on the most recent available imaging, and overall survival were reported as exploratory outcomes.

Given the small sample size and the absence of a control group, only descriptive statistics were used, with continuous variables reported as median (range) or mean (range) and categorical variables as count (%).

## 3 Results

### 3.1 Patient characteristics

We retrospectively analyzed seven patients (eight treatment sites) who underwent LRT between February and November 2025. The cohort comprised six men and one woman, with a median age of 69 years (range 57-77) and a median ECOG performance status of 2 (range 1-3). One patient received LRT at two distinct sites. The treated sites included: hepatocellular carcinoma with left adrenal metastasis; tongue squamous cell carcinoma with cervical lymph node metastasis; extraskeletal osteosarcoma with left psoas and pelvic metastases (the psoas lesion was a previously irradiated site); gallbladder carcinoma with abdominal lymph node metastasis; hypopharyngeal carcinoma with left cervical lymph node metastasis; lung adenocarcinoma with metastasis to the right femur; and recurrent ureteral carcinosarcoma with pelvic metastasis (a previously irradiated site). Baseline characteristics are summarized in **Table 1**.

### 3.2 LRT dosimetric parameters

The median maximal tumor diameter was 8.6 cm (IQR 7.4-9.9 cm), and the median GTV volume was 308 cc (IQR 265.8-461.7 cc; range 101.8-792.4 cc). The median lattice volume (V-Lattice) was 6.7 cc (IQR 3.5-10.8 cc), the median Ablation Dose Ratio (ADR) was 2.4% (IQR 1.7-2.7%), the median number of lattice vertices was 5 (IQR 3-9), and the median LDI was 1.6% (IQR 1.2-2.1%). The median peak-to-valley dose ratio (PVDR) was 2.9 (IQR 2.6-4.5). The median LTV dose per fraction was 12 Gy (range 6–15 Gy). Among the five sites that received combined EBRT, the mean total EBRT dose was 44.5 Gy (range 30–54 Gy); the remaining three sites received LRT alone. For combined-therapy sites, EBRT was initiated after a 2-day interfraction interval following the first LRT fraction. Prescription doses and dosimetric parameters are detailed in **Table 3**.

**Table 3:** Lattice radiotherapy (LRT) dosimetric parameters for the 8 treatment sites.

No.	Treatment site	MTD (cm)	V-GTV (cc)	V-Lattice (cc)	LTV/GTV Rx	EBRT Rx	D-Lattice (cm)	N-Lattice	LDI (%)	PVDR	ADR (%)
1	Left adrenal metastasis	8.79	317	3.98	40 Gy/5F	49.6 Gy/16F	1.2	3	0.95	4.78	1.25
2	Right cervical LN metastasis	8.33	299	3.10	30 Gy/5F	50 Gy/25F	1.2	4	1.33	5.61	1.10
3	Left cervical LN metastasis	8.20	251.6	5.80	15 Gy/1F	54 Gy/18F	1.5	3	1.19	4.28	2.29
4-1	Pelvic metastasis	9.29	452.4	9.45	60 Gy/5F	0	1.5	9	1.99	2.60	2.10
4-2	Left lower abdomen met.	6.60	280	7.67	60 Gy/5F	0	1.0	6	2.14	2.85	2.74
5	Pelvic/RLQ metastasis	10.50	471	12.08	48 Gy/4F	0	1.5	11	2.34	2.54	2.56
6	Abdominal metastasis	5.20	101.8	2.79	15 Gy/1F	39 Gy/13F	1.5	2	1.96	2.92	2.74
7	Right thigh metastasis	14.50	792.4	28.47	24 Gy/2F	30 Gy/10F	1.5	9	1.14	2.42	3.59

MTD, maximal tumor diameter; V-GTV, gross tumor volume; V-Lattice, total lattice (vertex sphere) volume; LTV/GTV Rx, prescribed lattice dose and fractionation; EBRT Rx, prescribed external beam radiotherapy dose and fractionation (0 indicates LRT alone); D-Lattice, vertex sphere diameter; N-Lattice, number of vertex spheres; LDI, lattice density index = (N-Lattice/V-GTV) X100%; PVDR, peak-to-valley dose ratio (ratio of mean peak dose to mean GTV dose); ADR, ablation dose ratio (proportion of GTV receiving the ablative-dose hotspot); LN, lymph node; RLQ, right lower quadrant; F, fraction.

### 3.3 Clinical outcomes

**Figure 2:**
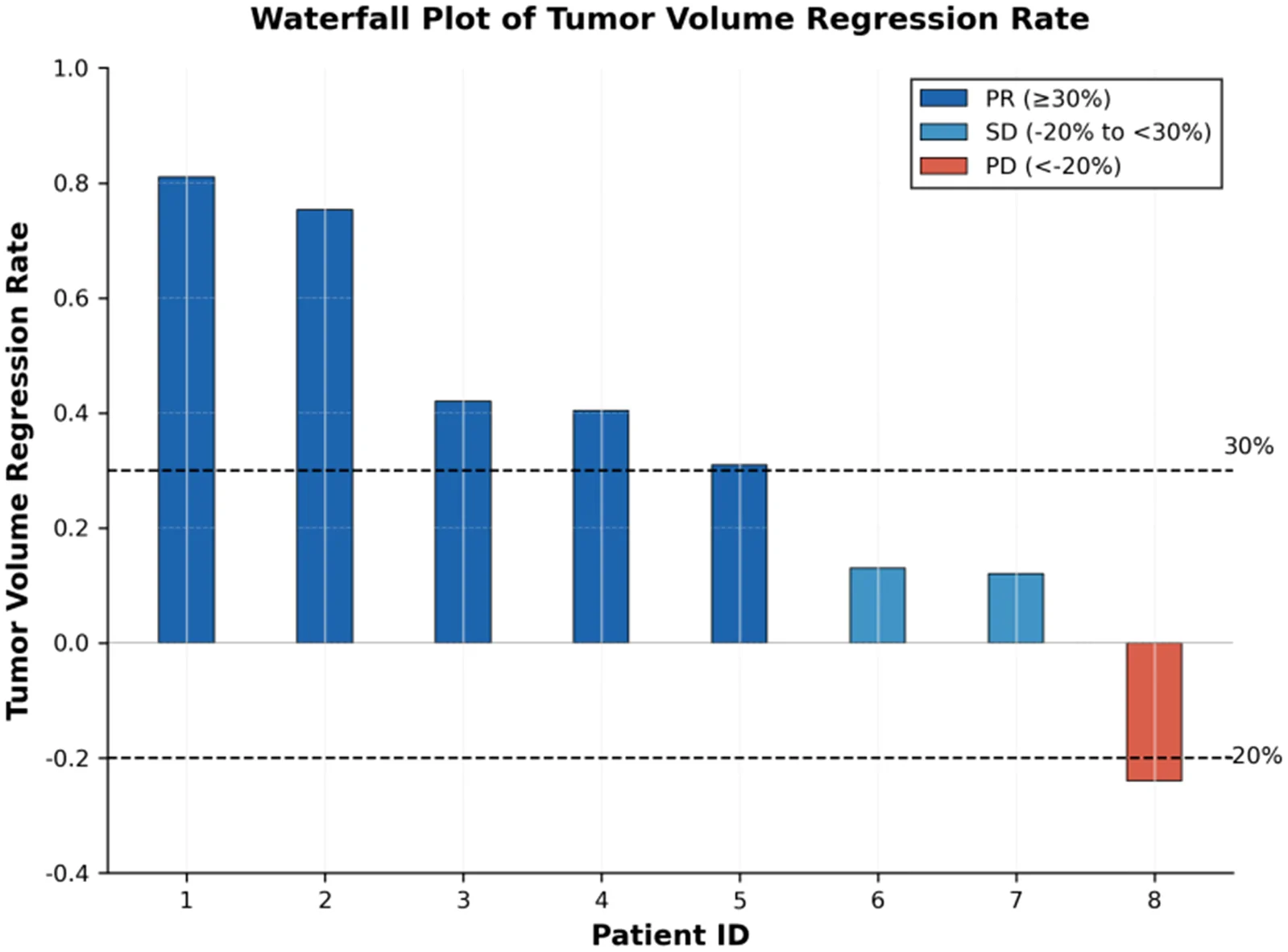
Tumor volume reduction rate after LRT.

**Figure 3:**
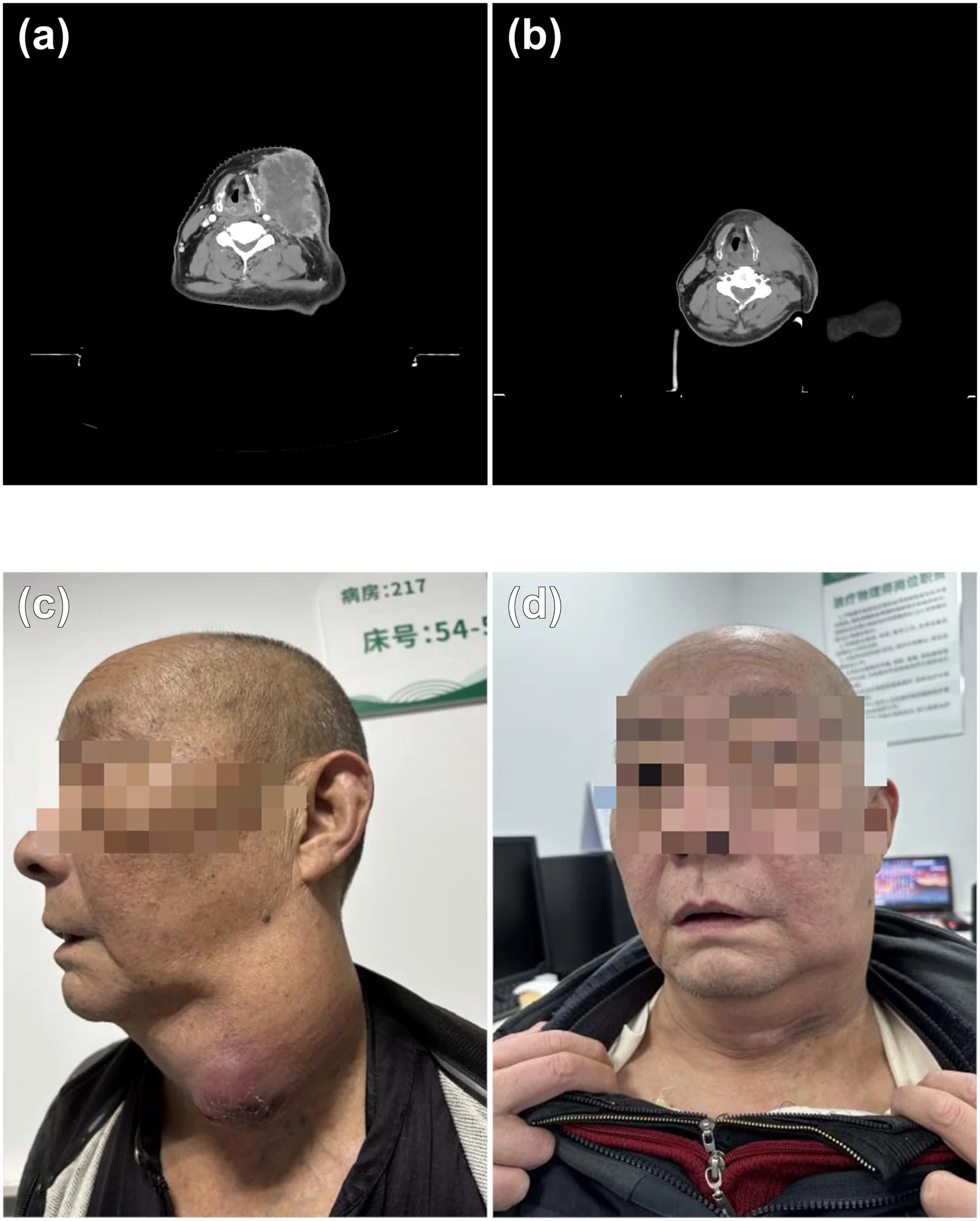
Tumor changes in a patient with hypopharyngeal carcinoma and cervical lymph node metastasis before and after treatment with LRT combined with EBRT. **(a, b)** Corresponding pre-treatment and 3-month post-treatment axial CT images at the same anatomical level. **(c, d)** Schematic representation (or clinical photograph) of the tumor appearance before treatment and 3 months after treatment.

All patients completed radiotherapy as planned. Per RECIST v1.1, PR was achieved in 5 of 8 sites (62.5%): HCC adrenal metastasis (P1), tongue SCC (P2), hypopharyngeal SCC (P3), gallbladder carcinoma abdominal metastasis (P6), and lung adenocarcinoma femur metastasis (P7). SD was observed in 2 sites (25.0%): renal extraskeletal osteosarcoma P4–1 and ureteral carcinosarcoma P5. PD occurred in 1 site (12.5%): renal extraskeletal osteosarcoma P4-2 (abdominal metastasis), with no CR observed. The objective response rate (ORR = CR+PR) was 5/8 (62.5%) and the disease control rate (DCR = CR+PR+SD) was 7/8 (87.5%). Tumor Reduction Rate (TRR), as illustrated in the waterfall plot (**Figure 2**), was >=30% in the 5 PR sites and between -20% and 30% in the 2 SD sites. Representative imaging is shown in **Figure 3**.

In a representative case, PET-CT performed one month after treatment of the hepatocellular carcinoma adrenal metastasis showed markedly reduced intralesional metabolism (SUV 2.5), central necrosis, and sparse tracer distribution. One patient with gallbladder carcinoma and abdominal lymph node metastasis, who presented with symptoms of gastric-outlet obstruction from antral compression, developed coffee-ground emesis two days after a single LRT fraction; symptoms resolved within two days of supportive care without melena, and the obstructive symptoms subsequently improved, permitting a liquid diet.

Treatment was well tolerated overall, with no grade >=3 toxicities. Pain and compressive symptoms improved to varying degrees. The symptom relief rate was 75% (6 of 8 sites). As of last follow-up, three patients remained alive and event-free, while four had died - one from tumor progression and three from non-oncological causes. Treatment responses and toxicities are summarized in **Table 1**.

## 4 Discussion

Advanced bulky tumors are often not suitable for surgical resection owing to their large volume and local invasion of surrounding tissues, while intratumoral liquefaction, necrosis, and hypoxia reduce sensitivity to conventional radiotherapy and systemic therapy. Spatially Fractionated Radiation Therapy (SFRT) is primarily applied in the palliative and definitive treatment of large-volume tumors (8, 23, 24). Its biological rationale is mainly related to the bystander effect, immune modulation, and microvascular effects (25–27): SFRT produces a highly non-uniform dose distribution that induces signaling-molecule release from irradiated cells and intercellular communication, while the preserved low-dose valley regions may maintain micro-structural pathways that permit immune-cell infiltration and the activation of systemic anti-tumor immune responses. Because of these mechanisms, the biological advantages of SFRT are considered more pronounced in large, radioresistant tumors than in conventional uniform-field radiotherapy.

Lattice radiotherapy can be delivered as lattice-vertex (LTV) irradiation alone, concurrent LTV and GTV irradiation, or sequential LTV and GTV irradiation, and standardized protocols are still lacking. The present study included all three modalities. Two patients with head-and-neck squamous cell carcinoma achieved complete tumor regression: one received concurrent lattice and conventional radiotherapy (lattice 30 Gy/5 F, once weekly, combined with conventional radiotherapy 50 Gy/25 F), and the other received one cycle of TP induction chemotherapy followed by sequential lattice and conventional radiotherapy (lattice 15 Gy/1 F, followed by conventional radiotherapy 54 Gy/18 F after a 2-day interval). Multiple studies of LRT in head-and-neck tumors have reported the highest response rates for squamous cell carcinoma: Mohiuddin et al. (28) reported a 94% response rate among 35 patients with squamous cell carcinoma in their classic analysis stratified by tumor site and histology, and Choi et al. (29) reported a 75% response rate in patients with squamous cell carcinoma receiving >75% EBRT dose in their IFRT study for large head-and-neck tumors. Although our sample is limited and follow-up short, our head-and-neck squamous cell carcinoma response rates are consistent with these reports.

This study also included radiotherapy for one case of primary hepatocellular carcinoma and one case of gallbladder carcinoma with abdominal metastasis. The patient with primary hepatocellular carcinoma received concurrent bevacizumab and a PD-1 inhibitor during radiotherapy; the adrenal metastasis (volume 317 cc) was treated with LRT at 40 Gy/5 F (once weekly) combined with conventional radiotherapy 50 Gy/25 F, and at 6 months the metastasis had shrunk by 40%, demonstrating the feasibility of this modality and dose design. The gallbladder carcinoma abdominal metastasis was treated with sequential LRT and conventional radiotherapy (lattice 15 Gy/1 F; conventional 39 Gy/13 F); after treatment the obstructive symptoms were relieved and the tumor regressed by 30% at 2 months. Mohiuddin et al. (28) reported an overall response rate of 94% when the LTV dose exceeded 1, 500 cGy versus only 62% for GRID doses below 1, 500 cGy (p=0.002), and patients receiving combined GRID plus external beam doses >=4, 000 cGy had a higher complete response rate than the low-dose group (24% vs 8%, p=0.06). In the present study, both hepatobiliary cases achieved an objective tumor response with palliative intent, and no grade >=2 gastrointestinal reactions occurred, consistent with the literature.

The remaining three LRT sites had sarcoma pathology and did not reach a partial response. For recurrent unresectable sarcoma, Mohiuddin et al. (10) reported a 76% response rate in 33 patients (40 sites) treated with GRID combined with conventional radiotherapy, rising to 95% for patients receiving >50 Gy, while Mohiuddin et al. (28) reported that among patients receiving GRID therapy alone 86% achieved an overall response (0% complete response, 86% partial response). In the present study, the sarcoma sites received LRT alone at 12 Gy per fraction with a total dose of 48–60 Gy. The failure to achieve a partial response may reflect multiple disease recurrences, extra-field metastases, the absence of combined conventional radiotherapy, large tumor volumes (approximately 450 and 470 cc), and a history of prior radiotherapy at two of these sites. Sarcoma is relatively radioresistant and may require higher-dose intensified treatment; these factors should be interpreted with caution given the very small numbers.

Of the seven patients, five received systemic therapy concurrently with LRT. Regimens were selected according to current CSCO and NCCN guidelines for each histology. Among those who received concurrent systemic therapy, PR was observed in three patients (P1, HCC; P3, hypopharyngeal SCC; P7, lung adenocarcinoma), SD in one (P4-1, renal extraskeletal osteosarcoma), and PD in one (P4-2, renal extraskeletal osteosarcoma), yielding an ORR of 3/5 (60%) and a DCR of 4/5 (80%). In the group that did not receive concurrent systemic therapy, both patients achieved PR (P2, tongue SCC; P6, gallbladder mucinous adenocarcinoma), corresponding to an ORR and DCR of 2/2 (100%).No grade ≥3 radiation-related adverse events occurred in any patient, and no obvious drug-related adverse reactions were observed during concurrent chemotherapy, immunotherapy, or targeted therapy. These findings are consistent with multiple studies reporting the safety of LRT combined with chemotherapy and immunotherapy for large tumors (30–33), indicating that LRT can be safely combined with contemporary chemotherapy, immunotherapy, targeted therapy, and antibody-drug conjugate (ADC) platforms. Notably, both patients who received LRT alone—without any concurrent systemic therapy—achieved partial response, demonstrating that the ablative vertex doses of LRT alone can produce a cytoreductive effect independent of systemic therapy. This observation is clinically important: it suggests that LRT remains effective in patients who are not candidates for chemotherapy, while also providing a rationale for combining LRT with systemic therapy in patients with better performance status.

A central technical aim was to determine whether VMAT-based LRT can be implemented using the conventional FF mode of a standard linear accelerator. Conventional GRID therapy is classically delivered with a flattened beam, and several high-volume centers explicitly do not use FFF modes for grid treatments (34). In contrast, contemporary VMAT-lattice delivery has predominantly employed FFF beams to leverage higher dose rates and shorter beam-on times (15, 35–38). However, the principal differences between FF and FFF beams lie in dose rate and delivery speed rather than in dose gradient quality. In the present series, all eight FF-mode plans met the acceptance criterion of a Gamma passing rate ≥95% (3%/2 mm). Measured beam-on time per fraction ranged from 4 minutes 15 seconds to 23 minutes 44 seconds (median, 9 minutes 31 seconds), which is clinically acceptable. We do not claim that FF-mode lattice delivery is novel; rather, our data indicate that FF-mode VMAT lattice represents a technically viable alternative in centers equipped only with standard FF linacs. However, the longer beam-on time necessitates careful assessment of patient cooperation, particularly in the frail ECOG 3 population included in this cohort.

For patients who are elderly, with advanced tumor stage, high tumor burden, and poor performance status, conventional radiotherapy regimens are often not feasible. The present study addresses this clinical challenge by comprehensively evaluating each patient’s functional status and clinical circumstances, and tailoring an individualized treatment strategy centered on LRT according to tumor imaging and biological characteristics. Our preliminary experience suggests that a patient-tailored LRT approach - integrating tumor volume, location, prior treatment, and performance status - can facilitate radiotherapy delivery in a challenging patient population often ineligible for standard curative-intent regimens.

### 4.1 Limitations

Several limitations should be acknowledged. First, this is a single-center, single-arm, retrospective analysis of only 7 patients (8 sites), and the treated tumors comprised a heterogeneous mix of histologies (squamous cell carcinoma, hepatocellular carcinoma, gallbladder carcinoma, adenocarcinoma, osteosarcoma, and carcinosarcoma) and anatomic sites; the reported symptom relief and disease-control rates therefore represent exploratory signals rather than generalizable efficacy estimates, and no control group is available for comparison. Second, symptom relief was a physician-adjudicated, retrospectively assessed endpoint based on NRS pain scores and clinical records; although the NRS is a validated instrument, it was not applied with a single uniform prospective protocol across all patients, and no patient-reported quality-of-life instrument was used. Third, the follow-up is relatively short (median 8 months, range 4–12 months), which is sufficient to capture acute toxicity and early disease control but precludes any definitive conclusion on long-term control, late toxicity, or overall survival. Fourth, the LRT dose, fractionation, and EBRT sequencing were individualized, which precludes a uniform prescription and limits inter-patient comparability. Fifth, because the cohort is small and heterogeneous, subgroup comparisons (e.g., by histology or treatment group) are descriptive only and lack statistical power. These limitations notwithstanding, the consistent technical feasibility (Gamma pass rate >=95% for all plans) and the favorable acute-toxicity profile provide a practical rationale for future prospective studies with standardized symptom-assessment instruments, predefined dose-fractionation schedules, and larger, more homogeneous cohorts.

## 5 Conclusion

In summary, this preliminary single-center study indicates that individualized VMAT-based lattice radiotherapy can be technically and safely delivered using a standard flattening-filter (FF) linear accelerator in patients with advanced bulky solid tumors, including those with poor performance status (ECOG up to 3). In this small, heterogeneous cohort, the approach showed a favorable acute toxicity profile (no grade >=3 events) and encouraging early symptom palliation and disease stabilization. These findings should be regarded as exploratory: the absence of a control group, the small and histologically diverse sample, the physician-adjudicated symptom assessment, and the short follow-up preclude any definitive conclusion on efficacy. Rather than claiming priority, our data add a fully verified FF-mode VMAT-lattice dataset to a limited evidence base and offer practical reference values for radiotherapy centers equipped only with standard FF linacs. Prospective studies with standardized symptom-assessment instruments, predefined dose-fractionation schedules, and larger, more homogeneous cohorts are warranted to confirm these signals and to define the optimal role, dose, and sequencing of FF-mode LRT.

## Data Availability

The raw data supporting the conclusions of this article will be made available by the authors, without undue reservation.
